# Therapeutic Potential of Saffron (*Crocus sativus* L.) in Ischemia Stroke

**DOI:** 10.1155/2021/6643950

**Published:** 2021-03-02

**Authors:** Shakiba Azami, Zahra Shahriari, Samira Asgharzade, Tahereh Farkhondeh, Mahmood Sadeghi, Fatemeh Ahmadi, Mohammad Mahdi Vahedi, Fatemeh Forouzanfar

**Affiliations:** ^1^Student Research Committee, Mashhad University of Medical Sciences, Mashhad, Iran; ^2^Medical Toxicology Research Center, Faculty of Medicine, Mashhad University of Medical Sciences, Mashhad, Iran; ^3^Cellular and Molecular Research Center, Basic Health Sciences Institute, Shahrekord University of Medical Sciences, Shahrekord, Iran; ^4^Medical Toxicology and Drug Abuse Research Center (MTDRC), Birjand University of Medical Sciences, Birjand, Iran; ^5^Department of Clinical Sciences, Faculty of Veterinary Medicine, Ferdowsi University of Mashhad, Mashhad, Iran; ^6^Department of Pharmacology, Faculty of Medicine, Zahedan University of Medical Sciences, Zahedan, Iran; ^7^Neuroscience Research Center, Mashhad University of Medical Sciences, Mashhad, Iran; ^8^Department of Neuroscience, Faculty of Medicine, Mashhad University of Medical Sciences, Mashhad, Iran

## Abstract

Stroke is the second leading cause of death and a main cause of disability worldwide. The majority (approximately 80%) of strokes are ischemic. Saffron (*Crocus sativus* L.) has been considered for medicinal purposes since ancient times. Pharmacological effects of saffron are attributed to the presence of crocin, crocetin, picrocrocin, and safranal. In the present review, we summarized the reported neuroprotective effects of saffron and its active constituents against cerebral ischemia stroke. Saffron and its components exert its beneficial effects as an antioxidant, anti-inflammatory, and antiapoptotic agent though inhibition of biochemical, inflammatory, and oxidative stress markers. Taken together, this review indicates that saffron and its ingredients could be a potent candidate in the process of new drug production for the treatment of ischemia stroke.

## 1. Introduction

Stroke is the second leading cause of death and a major cause of long-term disability worldwide [1]. Over recent decades, the burden of stroke has been highly increased in many regions of the world and, particularly, in developing countries [2]. Notably, as societies are getting older, the demand for long-term care for such illnesses is rising [1]. Ischemic stroke accounts for the highest percentage of all strokes, approximately 80%. The remaining 20% of strokes are hemorrhagic in nature [2, 3]. Cerebral ischemic stroke is the interruption of blood flow to the brain resulting from blood vessel blockage by vascular thrombus formation [4, 5]. Ischemic stroke contributes to tissue damage, neurological deficits, impairment of motor function, epilepsy, and poststroke dementia and may lead to death [6–8]. To date, the number of efficient curative treatments for ischemic stroke is limited. Currently, thrombolytic therapy with tissue plasminogen activator is the most effective treatment, which must be administered within 4.5 hours from ischemic stroke onset [9, 10]. Medicinal plants are invaluable source for the discovery of new therapeutic agents for a wide variety of human ailments [11–13]. The complex pathogenesis of stroke and multifactorial effect of herbal drugs and their active ingredients may indicate the promising future of herbal medicine for stroke treatment. Neuroprotective, anti-inflammatory, antioxidant, antiapoptotic, and vascular protective properties of herbal drugs are believed to be efficacious in treating stroke [11, 14–16]. Saffron has been extensively used for many medicinal purposes such as a pain reliever and expectorant, as well as for treatment of inflammation, wounds, and abscesses in folk medicine [17, 18]. Recent studies showed the value of saffron and its components in ischemia stroke. Therefore, in the current study, we aimed to review the anti-ischemic activities of saffron.  Table 1 shows experimental studies assessing the effect of saffron and its bioactive ingredients on cerebral ischemic stroke.

## 2. Research Methodology

A comprehensive search of Embase, PubMed, Scopus, and Google Scholar databases was carried out. Different relative keywords including *Crocus sativus*, safranal, stroke, neuroprotection, herbal medicine, and crocin were searched. The search included the papers published up to August 2020.

## 3. Saffron

Saffron is a valuable dietary spice derived from the flowers of *Crocus sativus* (Iridaceae). Saffron has been known since a long time as an herbal medicine [18, 19]. Saffron is now likely to be such an agent that has aroused physicians' interest [20]. At present, modern pharmacological investigations have demonstrated that saffron and its ingredients have a lot of therapeutic effects such as anticancer [21], antimicrobial [22], antineuropathic pain [23], hypolipidemic [24], antidiabetic [25], antianxiety [26], antitussive [27], antiobesity [28], antitremor [29], hypotensive [30], anticonvulsant [31], antidepressant [32], and antiarthritis effects [33]. Also, gastric ulcer healing [34], memory improvement [35], and management of metabolic syndrome [36] have been reported for this medicinal plant.

### 3.1. Bioactive Principles of Saffron

The main ingredients of saffron are water, nitrogenous matter, anthocyanins, glycosides, monoterpenes, aldehydes, flavonoids, vitamins (especially riboflavin: 56–138 *μ*g/g and thiamine: 0.7–4 *μ*g/g), volatile oil, proteins, amino acids, carbohydrates, minerals, raw fibers, and gums. Furthermore, picrocrocin (responsible for the bitter taste) and apocarotenoids ingredients such as crocetin, crocin (responsible for the color), and safranal (responsible for odor and aroma) are considered to be the main bioactive constituents [37, 38]. High-quality saffron has approximately 30% crocins, 5 to 15% picrocrocin, and 2.5% volatile compounds, including safranal [39].

## 4. Experimental Studies Conducted on the Effects of Saffron on Ischemia Stroke

Zhong et al. utilized the ischemic rat model to explore the neuroprotective effects of saffron on late cerebral ischemia injuries. Sprague-Dawley rats were subjected to focal cerebral ischemia induced by middle cerebral artery occlusion (MCAO). Rats were then assigned to six groups: sham, MCAO, Edaravone (as a positive control), and saffron extract (30, 100, and 300 mg/kg for 42 days). Saffron effectively reduced the levels of IL-6 and IL-1*β* and expressions of glial fibrillary acidic protein (GFAP), neurocan, and phosphocan, quantified by ELISA and Western blot, respectively. It also reduced neurological problems and spontaneous movements as well as anxiety-like behaviors and cognitive impairment, examined by elevated plus maze (EPM), marble burying test (MBT), and novel object recognition test (NORT). Besides, saffron showed anticerebral ischemia properties by reducing the infarct volume and glial scar formation [20].

Abdel-Rahman et al. designed a study to examine the role of vascular endothelial growth factor (VEGF) in the neuroprotective effect of saffron against cerebral ischemia/reperfusion injury (IR) in rats. Focal cerebral ischemia was induced by left MCAO. Wistar rats received only normal saline or saffron at doses of 100 and 200 mg/kg. Saffron was administrated intraperitoneally three weeks before surgery and then administered four times (60 min before surgery, during the surgery, and on 1 day and 2 days post-IR). A significant reduction in the latency to move their bodies and to fall in animals treated with saffron in relation to the control group was observed. According to the findings of this study, reducing oxidative stress and apoptotic proteins and exertion of vascular protection are among the main mechanisms mediating the neuroprotective effect of saffron. Also, the modulation of VEGF by saffron was considered as a pathway arbitrating its neuroprotective and antiapoptotic properties [40].

## 5. Experimental Studies Conducted on the Effects of Crocin on Ischemia Stroke

In one study, the anti-ischemic effect of crocin in male rats, which underwent a two-hour right MCAO, was investigated. Male rats were randomly divided into four groups: the sham-operated group, the MCAO group, and the crocin groups that were administrated 50 and 100 mg/kg of crocin orally. The treatments were applied for seven days, and rats were operated two h after the last administration. The results showed that the infarction areas and the neurological score decreased in crocin groups. Besides, crocin reduced the level of autophagy following cerebral ischemia by activating mTOR, which is a downregulator of autophagy processes. The results obtained from in vitro study showed that oxygen-glucose deprivation/reoxygenation significantly enhanced the proportion of apoptotic cells. Moreover, transmission electron microscope images demonstrate that the amount of autophagosome increased after oxygen-glucose deprivation/reoxygenation but decreased after treatment with 50 *μ*M crocin [41].

Huang and Jia examined the neuroprotective effect of crocin in the mouse model of hypoxic-ischemic encephalopathy. C57BL/6J mice were subjected to left common carotid artery ligation, and, after one-hour recovery, mice were treated by either hypothermia, crocin (10 mg/kg), or combined treatment. Results displayed that crocin treatment alone decreased brain damage and inflammation after hypoxia-ischemia. Combined treatment of crocin and hypothermia demonstrated increased therapeutic effect compared with single treatment, resulting in markedly less brain injury, decreased oxidative and inflammatory responses, and improved functional outcome. Their result demonstrated the beneficial effect of crocin as a part of combined therapy to enhance the neuroprotective effect of hypothermia and reduce hypoxia [42].

The therapeutic effect of crocin in improving the blood-brain barrier (BBB) disruption following MCAO was evaluated. 24-month-old rats randomly received either vehicle (controls) or crocin (10, 20, 40, or 60 mg/kg) every alternate day for two months before ischemia induction. In the presence of cerebral ischemia, crocin saved the BBB function. Besides, loss of tight junction complexes, the activity of matrix metallopeptidase (MMP), and increased NADPH oxidase were all attenuated in the crocin-treated group. These findings revealed that the antioxidant capacity of crocin could ameliorate the ischemia-induced damage [43].

Another study showed that crocin pretreatment (40 mg/kg for ten days) once daily attenuated apoptosis, probably mediated by decreasing oxidative stress index and increasing total antioxidant capacity (TAC) induced by reactive oxygen species (ROS) generation and inhibiting the protein expression of HIF-1*α*, TUNEL, and caspase-3 in a rat global cerebral IR induced by bilateral common carotid artery occlusion (BCCAO), followed by 30-minute reperfusion [44].

Crocin treatment (30, 60, and 120 mg/kg) markedly reduced infarct volume by 64%, 74%, and 73%, respectively, in a rat model of transient focal cerebral ischemia. Indeed, the most effective dose of crocin was 60 mg/kg and suggested that higher doses do not have more protective effect. Administration of crocin (60 mg/kg) 1 hour before, at the start, or 1 hour after ischemia decreased brain edema by 48%, 52%, and 51%, respectively. Furthermore, crocin (60 mg/kg) markedly decreased the level of malondialdehyde (MDA) and enhanced activity of glutathione peroxidase (GPx) and superoxide dismutase (SOD) in the ischemic cortex [45].

Using an MCAO rat model, Sarshoori et al. explored the histopathological manifestations of transient focal cerebral ischemia in response to crocin intraperitoneal injection. Briefly, Wistar rats were treated by intraperitoneal injection of 50 and 80 mg/kg of crocin at the start of ischemia. A significant decrease in infarct volume was evident in the cortex and striatum at the right hemispheres of the crocin group 24 hours after ischemia. However, 80 mg/kg of crocin exerted a higher efficacy. Another histopathological finding was that crocin effectively reduced axonal fragmentation, fiber demyelination, and prenecrotic neurons number in the ischemic areas. This study concluded that crocin is sufficient to suppress ischemia-induced damage by preventing the alternations of histopathological parameters [46].

Transient global cerebral I/R markedly promoted the generation of nitric oxide (NO) and the activity of nitric oxide synthase (NOS). The reperfusion led to serious edema with mitochondrial injuries in the cortical microvascular endothelial cells, as well as increased membrane G protein-coupled receptor kinase 2 (GRK2) expression, and decreased cytosol GRK2 expression. Besides, increased phosphorylation of extracellular signal-regulated kinase1/2 (ERK1/2) and reduced expression of MMP-9 were detected in cortical microvessels after I/R (20 min/24 h). As well as the positive control crocin (20 mg/kg, 21 days), pretreatment with Weinaokang, which contains active compounds of Ginkgo, Ginseng, and saffron, on ischemic injuries (20, 10 mg/kg, 21 days) significantly inhibited nitrative injury and modulated the ultrastructure of cortical microvascular endothelial cells. Besides, Weinaokang inhibited GRK2 translocation from cytosol to the membrane (at 20 mg/kg) and decreased MMP-9 expression and ERK1/2 phosphorylation in cortical microvessels [47].

Zhang et al. [48] showed that oral administration of crocin had better effectiveness in cerebral protection than an intravenous injection. Crocin and its metabolite crocetin were not detected in the brain of cerebral I/R rats, indicating a target site of periphery. After oral administration of crocin instead of intravenous injection, abundant crocetin was found in plasma. Indeed, oral administration of crocetin displayed similar cerebral protection to that of crocin, but this effect was not clearly found by intravenous crocetin administration, representing the importance of crocetin in gut. Furthermore, orally administered crocin demonstrated less cerebral-protective effect in pseudo-germ-free MCAO rats.

Among saffron's ingredients, crocin was the most effective in promoting mRNA expression of gamma-glutamylcysteine synthase, which contributes to glutathione synthesis as the rate-limiting enzyme, and the carotenoid markedly decreased infarcted areas caused by MCAO in mice [49].

## 6. Experimental Studies Conducted on the Effects of Safranal on Ischemia Stroke

Sadeghnia et al. utilized the rat models of transient MCAO, which continued by 24 hours of reperfusion. Safranal (72.5 and 145 mg/kg) was administrated intraperitoneally by 0, 3, and 6 hours after reperfusion. Compared with the control animals, the level of MDA significantly decreased in safranal groups, while total sulfhydryl (SH) and antioxidant power showed a considerable increase. The percentages of degenerated hippocampal cells, mean infarct volume, and neurological deficits were reduced with both doses of safranal. Collectively, safranal exerted an explicit neuroprotective effect on transient focal cerebral ischemia [50]. One study showed that administration of safranal increased the total SH contents and antioxidant capacity in hippocampus of rats with transient global cerebral ischemia. Besides, administration of safranal reduced the MDA level in hippocampus of rats with transient global cerebral ischemia [51].

In a recent study by Forouzanfar et al. [52], oxygen-glucose deprivation exposure decreased the cell viability and enhanced intracellular ROS production, apoptosis, and oxidative DNA damage, in comparison with untreated control PC12 cells. Pretreatment with safranal (40 and 160 *μ*M) markedly attenuated cell death, apoptosis, and oxidative damage induced by oxygen-glucose deprivation in PC12 cells. Additionally, safranal significantly decreased the overexpression of Bax/Bcl-2 ratio and active caspase-3 following oxygen-glucose deprivation.

## 7. Human Studies Conducted on the Effects of Saffron on Ischemia Stroke

Fifty ischemic stroke patients with specific severity of symptoms at admission (National Institute of Health Stroke Scale (NIHSS) score = 5–20) and less than 24 h since the onset of stroke were randomly assigned to receive either routine stroke care (control group) or routine care plus aqueous extract of saffron capsule (200 mg/day) (saffron group). Patients in both groups were monitored for the study days including four days of hospital stay and the following three months. In saffron group, the severity of stroke during the first four days was reduced compared to the control group. Compared to the concentrations on the first day, serum neuron specific enolase and s100 levels were markedly reduced and brain-derived neurotrophic factor (BDNF) level was enhanced in the saffron group on the fourth day. At the end of the three-month follow-up period, the mean Barthel index was markedly higher in the saffron group compared to the control group [53].

A recent study has been conducted to evaluate the role of aqueous extract of saffron in decreasing oxidative stress among patients with ischemic stroke. Forty ischemic stroke patients with NIHSS score = 5–20 less than 24 h since the onset of stroke were randomly divided into two groups: control group and saffron group. The patients in control group received routine stroke care and the patients in saffron group received routine care plus capsule of saffron 400 mg/day (200 mg twice daily) over a period of four days. On the fourth day after the onset of ischemic stroke, the saffron-treated group had higher levels of antioxidant enzymes activities and GSH and TAC levels than the control group, while MDA concentration was lower. Furthermore, the severity of stroke was markedly decreased after 4 days in the saffron-treated group. The severity of stroke was negatively associated with the GSH and TAC concentrations and positively associated with the MDA concentration [54].

## 8. Possible Mechanism of Neuroprotective Potency of Saffron and Its Ingredients in Ischemia Stroke

Several investigations attempted to clarify the mechanisms underlying the neuroprotective function of saffron and its active derivatives in ischemia stroke. Oxidative stress is caused by an imbalance between prooxidants and antioxidants, resulting in overproduction of ROS. ROS are biphasic and are involved in natural physiological processes as well as in a number of disease processes, thereby mediating damage to cellular structures such as lipids, membranes, proteins, and DNA [55, 56]. The cerebral vasculature is a main target of oxidative stress which plays a major role in the pathogenesis of ischemic brain injury after cerebrovascular attack [55, 56]. Consequently, oxidative stress contributes directly to necrosis and apoptosis via a number of pathways in ischemic tissue [57, 58]. Previous studies showed that saffron exhibited a potential antioxidant and antiapoptotic property, which is attributed to the bioactive ingredients of saffron [40, 50, 59]. Brain edema, as a result of ischemic stroke, could be attributed to the elevated brain natriuretic peptide (BNP) level [60]. Saffron has exhibited a substantial effect on decreasing BNP in brain of I/R rats. It is suggested that saffron-induced BNP lowering may be due to its antioxidant properties [40].

Recently, it has been reported that saffron effectively enhanced the expression of VEGF in ischemic boundary zone, which plays a critical role in cerebral protection after I/R injuries [40]. Indeed, VEGF confers neuroprotection and promotes neurogenesis and cerebral angiogenesis [61]. Brain ischemia leads to an acute and prolonged inflammatory process, characterized by rapid activation of resident cells (mainly microglia), production of proinflammatory mediators, and infiltration of numerous types of inflammatory cells (including neutrophils, different subtypes of T cells, monocyte/macrophages, and other cells) into the ischemic brain tissue [62, 63]. Recent experiment has found that saffron could attenuate inflammation in the ischemic brain [20, 42].

## 9. Conclusion

Collectively, the data from the referred experiments provide insight into the beneficial effect of saffron and its derivatives, particularly safranal and crocin, against neural injuries in the cerebral ischemia. The studies indicated that administration of saffron or its ingredients could make a notable contribution to the prevention of histopathological alternation as well as improvement of neurological deficit. However, several investigations have noted that the downregulation of apoptosis, inflammation, and autophagy, alleviation of glial scar formation, prevention of oxidative stress, and reduction of brain edema are among the most mentioned mechanisms mediating the saffron efficacy. Thus, saffron and its active constituents can be a candidate as therapeutic agent, either alone or in a combined form, with current strategies for ischemia stroke treatment.

## Figures and Tables

**Table 1:** Experimental studies assessing the effect of saffron and its bioactive ingredients on cerebral ischemic stroke.

Product	Animal type	Stroke model	Dose administration	Duration of administration	Major effects	References
Saffron hydroalcoholic extract	Male Sprague-Dawley rat	MCAO	30, 100, and 300 mg/kg/day orally	2 h at the first day and once daily from day 2 to day 42 after ischemia	(1) ↓Body weight loss, neurological deficit, spontaneous activity, infarct volume, and glial scar formation.	[20]
					(2) ↓GFAP, neurocan, and phosphocan in ischemic boundary zone.	
					(3) ↓Contents of IL-6 and IL-1*β* in ischemic boundary zone.	
					(4) ↑Content of IL-10 in ischemic boundary zone.	

Saffron hydroalcoholic extract	Male Wistar rat	MCAO	100 and 200 mg/kg/day intraperitoneally	3 successive weeks before being subjected to left brain I/R and then administered four times (60 min before surgery, during the surgery, and 1 day and 2 days after the I/R)	(1) ↓Latency to move the body.	[40]
					(2) ↓MDA and NO in brain tissue.	
					(3) ↑VEGF in brain tissue.	
					(4) ↓BNP in brain tissue.	

Crocin	Adult male SD rats of specific pathogen-free (SPF) grade	MCAO	50 and 100 mg/kg/day orally	7 days before MCAO induction	(1) ↓Neurological score, infarct volume.	[41]
					(2) ↓p-AMPK/AMPK, LC3-II/I, and ULK1 in brain tissues.	
					(3) ↑p-mTOR/mTOR and p62 in brain tissues.	

Crocin	C57BL/6J mice	Hypoxic-ischemic encephalopathy	5, 10, and 20 mg/kg	Every 12 h, starting immediately or at 2 h after hypoxia-ischemia.	(1) ↓Tissue loss and brain infarction.	[42]
					(2) ↓iNOS and COX-2 mRNA expression in the brain tissues-neurological function recovery.	

Crocin	24-month-old male rat	MCAO	10, 20, 40, and 60 mg/kg, orally	Every two days for 2 months before induction of MCAO	(1) ↓Infarct volume.	[43]
					(2) ↑BBB integrity.	
					(3) ↓Loss of tight junction proteins.	
					(4) ↓Enhanced NADPH oxidase in brain tissues.	
					(5) ↓MMP-2 and MMP-9 level in brain tissues.	

Crocin	Adult female Sprague-Dawley rats	BCCAO	40 mg/kg/day orally	10 days before CCAO induction	(1) ↓Ischemic lesions.	[44]
					(2) ↓Hippocampal TUNEL-positive cells.	
					(3) ↑TAS in brain tissues.	
					(4) ↓OSI in brain tissues.	
					(5) ↓Caspase-3 and HIF-1*α* in brain tissues	

Crocin	Adult male Wistar rat	MCAO	15, 30, 60, and 120 mg/kg intraperitoneally	At the start and 1, 3, and 6 hours after MCAO induction	(1) ↓Neurological deficit and infarct volume.	[45]
					(2) ↑SOD, GPx, and TAC in the cortex of the brain tissue.	
					(3) ↓Brain water content.	
					(4) ↓MDA level in the cortex of the brain tissues.	

Crocin	Adult male Wistar rat	MCAO	50 and 80 mg/kg intraperitoneally	At the start of ischemia	(1) ↓Neurological deficit and infarct volume.	[46]
					(2) ↓Axonal fragmentation, fiber demyelination, and prenecrotic neurons number.	

Crocin	Male ddY mice	MCAO	10 mg/kg intravenously	Before and 3 hours after MCAO induction	↓Infarct volume.	[49]
Safranal	Adult male NMRI rat	Global cerebral ischemia was induced using the four-vessel occlusion method	727.5, 363.75, 145.5, and 72.75 mg/kg intraperitoneally	5 min prior to reperfusion and the administration was continued every 24 hours for 72 hours after the induction of ischemia	(1) ↓MDA level in brain tissues.	[51]
					(2) ↑Antioxidant capacity in brain tissues.	
					(3) ↑Total thiol concentration in brain tissues.	

**Safranal**	Adult male Wistar rat	MCAO	72.5 and 145 mg/kg intraperitoneally	0, 3, and 6 hours after induction of MCAO	(1) ↓Neurological score, infarct volume, and hippocampal cell loss.	[50]
					(2) ↓MDA level in brain tissue.	
					(3) ↑Antioxidant capacity in brain tissue.	

SD, Sprague-Dawley; MCAO, middle cerebral artery occlusion; BCCAO, bilateral common carotid artery occlusion; GFAP, glial fibrillary acidic protein; MDA, malondialdehyde; GPx, glutathione peroxidase; NO, nitric oxide; TAC, total antioxidant capacity; VEGF, vascular endothelial growth factor; BNP, brain natriuretic peptide; BBB, blood-brain barrier; ULK, Unc-51 like autophagy activating kinase; NADPH, nicotinamide adenine dinucleotide phosphate; MMP, matrix metallopeptidase; SOD, superoxide dismutase.

## Data Availability

No data were used to support this study.

## Supplementary Materials

Supplementary Materials Graphic abstract: saffron and its ingredients showed protective effect against ischemia stroke.
