# Supplementary material for: Therapeutic Potential of Saffron (Crocus sativus L.) in Ischemia Stroke
Source: Evid Based Complement Alternat Med. 2021 Mar 2;2021:6643950. doi: 10.1155/2021/6643950 (PMC7943274; doi:10.1155/2021/6643950)
Supplement: Supplementary Materials — Graphic abstract: saffron and its ingredients showed protective effect against ischemia stroke. [file 6643950.f1.docx]

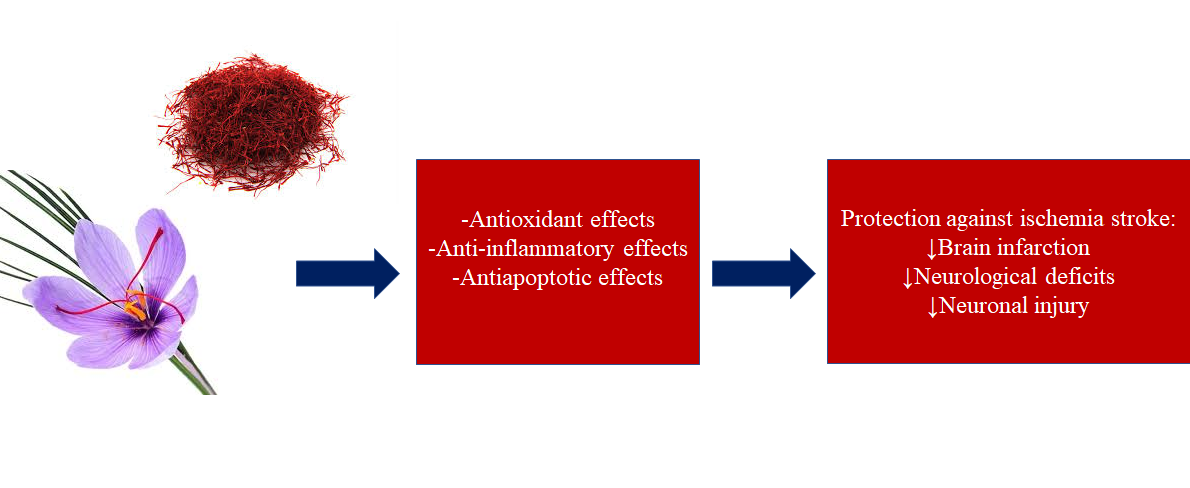


**Graphic abstract.** Saffron and its ingredients showed protective effect against ischemia stroke.
